# Use of crystal methamphetamine among male adolescents in Cape Town, South Africa: Caregivers' experiences

**DOI:** 10.1186/s13011-017-0102-9

**Published:** 2017-03-27

**Authors:** Kwaku Oppong Asante, Antonio G. Lentoor

**Affiliations:** 1 Department of Psychology, University of Ghana, P.O. Box LG 84, Legon, Accra Ghana; 2 Department of Psychiatry and Mental Health and Valkenberg Hospital, University of Cape Town, Observatory, Cape Town, South Africa

**Keywords:** Addiction, Crystal methamphetamine, Family disintegration, Mothers’ experiences, Psychological distress, Qualitative study

## Abstract

**Background:**

Against the background that crystal methamphetamine (colloquially known as “tik”) is extensively used by the emerging working class Coloured youth in Cape Town, South Africa, this exploratory qualitative study was conducted to explore the experience of mothers whose children use methamphetamine.

**Methods:**

The researchers conducted one-to-one semi-structured in-depth interviews with sixteen (16) purposively selected caregivers (mothers) whose sons use methamphetamine. Interviews were recorded and simultaneously translated and transcribed. Thematic analysis was used to identify themes related to the experiences of caregivers of youth with methamphetamine problems.

**Results:**

Findings showed that youth misbehaviour provided a context that led to feelings of shame and embarrassment. Participants also experienced personal challenges which included emotional problems, fear and self-blame. Participants also expressed family disruptions and financial drain as adverse experiences as a results of their sons’ misbehaviour.

**Conclusion:**

The study results highlight the psychosocial challenges for caregivers of children who use methamphetamine. These findings underscore the need for effort to be directed at the development of formal support interventions for mothers of youth who are troubled with addiction.

## Background

Crystal methamphetamine, the most widely used synthetic drug globally, has spread rapidly throughout the South African society [1, 2]. It is a highly addictive synthetic psycho-stimulant that increases energy and feelings of euphoria, among other physiological effects [3]. It is locally referred to as ““tik” due to the “ticking” sound produced when smoked [4]. The prevalence of methamphetamine (“tik”) use is highest in the Western Cape Province, and its impact has been felt within the Cape Flats over the past fifteen years [4, 5]. Methamphetamine is perceived as the fastest growing addictive substance ever introduced into the market, and at some point, has even exceeded commonly used drugs such as dagga, mandrax and ecstasy [6, 7].

Methamphetamine use within the South African context is regarded as a recent phenomenon that needs to be considered within a particular psychosocial context [4, 8]. South Africa is a country that is shaped by specific sociopolitical and historical factors which are directly linked to social inequalities as it still retains many negative characteristics of the old apartheid regime [4]. The Western Cape province of South Africa is a predominantly Coloured area and many of its communities grapple with high rates of ‘gangsterism’, drug abuse, crime and organised crime [9]. Compounding the problem is the existence of methamphetamine, which has rooted itself deep within the already affected social milieu of these communities that is already clouded by the impact of substance use [9–11].

Over the past decade, the emergence of methamphetamine has caused a new moral panic. Anecdotal evidence suggests there are over 200,000 tik users in and around the city of Cape Town, and about half the people receiving treatment cite tik as a primary or secondary substance of abuse—far ahead of alcohol [8]. Moreover, statistics indicate that 91% of teenage methamphetamine users were Coloured males, and that the average age of these users was 16 years old [6, 7]. Furthermore, in the Western Cape Province, on an average, one out of every five school-going youth is actively using crystal methamphetamine [12].

The challenge with substance abuse is that a high percentage of adolescents experiment with illegal mood-altering drugs at a young age and with highly addictive drugs such as methamphetamine [6]. Prior to the emergence of methamphetamine, cannabis was the most widely used drug among the urban populations [4]. A few studies have also indicated that 12 to 19 year old adolescents react severely to methamphetamine as it is a highly addictive drug [11]. It is also evident that methamphetamine is cheap, accessible and can be easily manufactured, making it the third most commonly reported primary illicit substance of abuse (after methaqualone and marijuana) [4, 13]. Moreover, the problem is not merely that it is so readily available, but also methamphetamine is the drug of choice for young people [13].

Methamphetamine use is regarded as a phenomenon that affects not only the individual but the community as a whole, as its use commonly results in the disintegration of family structures, where, in many cases, individuals drop out of schools and even end up dying by suicide [6, 7]. There is a great plight among parents who are striving to combat this drug that is destroying their children’s lives. Currently, most of these communities have limited governmental structures in place to fight the extensive abuse of methamphetamine. However, there have been certain initiatives from parents in some communities to create a campaign to deal with this social dilemma. In many instances, however, parents who seek help for their children with extensive methamphetamine use are consequently exposed to various psychological problems themselves [14]. Another gloomy phenomenon associated with methamphetamine use is that it even attracts individuals who have never experimented with drugs before [7].

The rapid and extensive spread of methamphetamine remains a problem in the South African context and is a significantly under-researched phenomenon. Anecdotal evidence of the impact of methamphetamine use on society, including some of the hard-hit communities [9, 13, 15] are well documented. Methamphetamine use has also been shown to be associated with childhood sexual abuse and HIV sexual risk behaviours including commercialisation of sex and having unprotected sex with multiple sexual partners [16, 17]. A recent study has shed some insight into what attracts the youth and how they are initiated into the use of methamphetamine [18]. However, little data is available to shed some insight into the impact of methamphetamine use on the caregivers of the users. The lived experiences of individuals whose relatives use methamphetamine are not well known, neither is the psychological effect it has on them. This exploratory qualitative study was therefore conducted to explore the lived experience of mothers whose children use crystal methamphetamine within a working-class community. Such knowledge is important to gain a better understanding of the psychosocial impact of the drug use locally, and to help develop formal support interventions for mothers of youths who are troubled with its addiction.

## Method

### Research design and setting

This study was qualitative in nature. This approach was suitable for the study, as it enabled the researchers to develop an understanding of social life and to discover the subjective meaning that people construct and attach to their actions. The study was conducted in a Coloured community on the Cape Flats, known as Lavender Hill, a township located 23.7 km from Cape Town’s city centre. The township was established between 1972 and 1974 and is currently home to over 32,598 residents; the majority are Coloured people with a few Black Africans. The terms ‘Black’ and ‘Coloured’ originated from the apartheid era and refer to demographic markers that do not necessarily signify inherent characteristics. The terms refer to people of African and mixed (African, European and/or Asian) ancestry, respectively. Lavender Hill lacked many essential resources, ranging from good housing to recreational resources, and was characterised with high levels of unemployment, poverty, violence, drug abuse and crime [12]. The 2011 City of Cape Town Census indicated that 42% of residents in the area were unemployed, and the majority of adults who were employed were unskilled and manual labourers [19].

### Sample and sampling approach

A group of 16 mothers who have had first-hand experience with their sons’ use of methamphetamine participated in this study. The participants were a homogenous group as all of them were Afrikaans speaking, lived in Lavender Hill community and shared the same socioeconomic status and ‘race’. The participants’ sons’ using methamphetamine were between the ages of 15 and 20 years, whereas the mothers’ ages ranged between 30 and 45 years. The majority (*n =* 10) of the participants were unemployed, half (*n =* 8) of them were married but five were married to husbands who were not their sons’ biological fathers (step-fathers) whilst the remaining half were single mothers. The number of children had by each participant ranged from two to five. Participants were included in the research through snowball sampling strategy, which allowed the researchers to locate participants by asking one key informant to identify individuals who have experienced a similar phenomenon. Participants were selected if they met the following inclusion criteria: 1) their son(s) had used methamphetamine for at least one year or had a history of methamphetamine use, 2) have experience with effect of tick directly through their son’s or indirectly through witnessing someone using it in the home or community.

### Procedure

Qualitative individual in-depth interviews were conducted by the second author (whose mother tongue is Afrikaans) at a convenient place with each participant in Afrikaans. One-to-one interviews with the mothers were separately conducted to allow each participant to discuss the topic freely and openly. A semi-structured interview schedule was used to guide the interview process which covered issues such as the context within which methamphetamine use takes place, the importance of familial relationships, community relationships and community response to drugs, the existence of support systems and the availability of resources, the culture of drug use within the wider community, issues of unemployment and the impact of methamphetamine on the individual and family. Some specific questions asked were: *How does your son’s use of methamphetamine affect your life? How do family and friends feel about your son’s behaviour due to his extensive use of methamphetamine? How did you feel when you found out that your child was extensively using methamphetamine?* The interview questions were developed based on review of previous studies and the authors’ understanding of the subject under investigation. All participants signed a written informed consent form prior to participation in the study. They also gave permission for the interviews to be audio-recorded. All the interviews were simultaneously translated verbatim into English by the second author so as not to lose the authenticity of meaning produced through the stories. The average duration of each interview was 40 min. The study was approved by the Faculty of Humanities Research Ethics Committee, University of Cape Town, South Africa. To ensure confidentiality of the participants, the names and location were not reported.

### Data analysis

The audio-recordings of the interviews were simultaneously translated and transcribed in English. The software NVivo 10.1 was used to structure the data and analysed thematically [20]. The six steps involved in thematic analysis [20] were followed to analyse the data: (1) familiarisation with the data; (2) generating initial codes; (3) searching for themes; (4) reviewing themes; (5) defining and naming themes; and (6) producing the report. A researcher with adequate knowledge in the subject area was engaged to cross-validate the emergent themes. Furthermore, constant engagements with the audio interviews occurred to ensure authenticity of coding. The themes were discussed by the authors and consensus was reached about the coding to reduce bias. These steps were aimed at enhancing the credibility of the findings.

## Results

Following the analysis of the data, the findings were organised into four major themes: (a) son’s misbehaviour, b) personal challenges, c) family disruptions and d) financial drain. The quotations illustrating the above-mentioned themes are illustrated in Table 1. Son’s misbehaviour describes adverse behaviours of the child which might influence the lifestyle of the caregiver. Here two themes were identified: 1) criminality/stealing; and 2) shame and embarrassment. Personal challenges reflect the psychosocial challenges that the caregiver had to face because of their sons’ extensive reliance on methamphetamine. Three main sub-themes were found, namely: 1) emotional problems, 2) fear, and 3) self-blame. Family disruptions describes the strained interpersonal relationships with family and community members due to their sons’ addiction, and financial drain describes the financial consequences as a result of methamphetamine use by their sons. To better capture the participants’ experiences, the local term for methamphetamine (“tik”) was used throughout the quotations.

**Table 1:** Themes and quotations illustrating the experiences of caregivers

Theme/Sub-themes	Quotations
*Son’s misbehaviour*	
Crime and stealing	If you are on “tik” you have to steal, you can’t get it free and then you have to steal for it and that is the quickest way to go to jail and prepare to die because they can shoot you when you break into a house…and that is what these “tik” users do-including my son, that’s why I fear him because he steals in the house and even becomes violent, wanting money where I do not have money because I do not work *(Participant 2).*
	He wasn’t working and was stealing from my house and breaking into the houses in the community and ended up getting arrested by the police (*Participant 8)*.
Shame and embarrassment	You feel embarrassed, it was so embarrassing, and I was hurt. To think I had to lock myself up in my own house because he steals everything he touches (*Participant* 11*)*
	I’ve lost friends because of his behaviour. He could go to my friends’ house, put on the kettle; he can make everything that he wants there, but not anymore. He and his friends broke into her house and stole things from her. It makes me feel so embarrassed and ashamed *(Participant 5).*
*Personal challenges*	
Emotional problems	I didn’t know what to expect at home. It’s frustrating to me. I go into a panic attack and stay there for so long, can’t breathe, my heart beats and stuff like that. I don’t wish to be at home but the fact is where must I go? I have to face it; I cannot take my problems to someone else. In that state, I become very nervous. I am telling you my nerves become very high. Sometimes I just feel like giving up, you know. I can’t take this anymore, but then I have to think about his brothers and sister. They are keeping me alive, only them *(Participant 2).*
Fears	I can expect anything to come to my front door. They could arrive one day knocking at my door and tell me that my child is dead or seriously injured, and he was lying in the hospital. I expected the worse. I was traumatised *(Participant 12)*.
	I fear for my son that he can be hurt because of his criminal and stealing behaviour. Since he got addicted to “tik”, his behaviour have been very erratic and frankly I am currently afraid that something bad may happen to him anytime soon *(Participant 1)*.
	I currently live in a state of fear as I don’t know what will happen to him tomorrow. His violent and criminal behaviour make me worry and constantly afraid of what will happen next *(Participant 7)*.
Self-blame	You know sometimes I feel, as though I am to blame. I always ask myself this question. Did I over protect him? But I feel that I did what was expected of me as a mother but I now feel like I am responsible for what he has grown up to be now. I feel as I have failed as a mother *(Participant 10)*.
	I gave my child everything I could but maybe I don’t think it was enough. I think I gave him the best but on the other hand, I spoiled him, yeah, I spoiled him too much. I felt very hurt. Somewhere I failed as a mom *(Participant 2)*.
*Family disruptions*	My spouse and I are constantly fighting; you see because he does not understand what “tik” does to my child. He is not my son’s father but he raised him, gave him everything a father can. I know he [the son] is disrespectful to my spouse you see, and he was never like that, not until he started using the “tik” *(Participant 4)*.
	My whole family is affected by “tik”. It affected the family. It’s disrupted the entire family…it has caused chaos (Participant 15).
	It’s [her son’s behaviour] affecting me and obviously, I know it’s affecting all my children, all of them, all of them. He is aggressive in the house and my husband [son’s stepfather] is getting sick and tired. He doesn’t even respect him anymore (Participant 11).
*Financial drain*	I had to stop working because he cannot care for himself…and due to his criminal behaviour he ended up in front of the magistrate. I never had to attend court cases as mush as I do now. He already has one court case he refused to go…many times I had to stay at home and not go to work and as result I was fired from my employment (Participant 9).
	He is not capable of working as he is unable to hold on to his job. At times, he just works for a day and uses the money for “tik”. Ever since the “tik” story came out, I don’t even receive money [from him]. At one point, he only pays me R20 [$1.30]. But prior to getting addicted to “tik”, he used to work one day for me and the next day for himself. He doesn’t do that anymore (Participant 15).

### Sons’ misbehaviour

The majority of the participants revealed that their children’s behaviour was a problem for them. According to these participants, their sons’ involvement in criminal activities such as breaking into houses and stealing of items was a problem to them. Some of the participants feared that their children could be killed as a result of the misbehaviour. Stealing and petty theft are common among young adults addicted to methamphetamine [14, 21]. Society regards such behaviours as unacceptable and dangerous to community safety. It was therefore not surprising that some of the participants reported that their children had sometimes been arrested by the police for such criminal activities. The fear of attack by their own children, should they not be able to give or provide what they want from them, was also revealed by the participants.

The consequences of the sons’ misbehaviour are the shame and embarrassment that the caregivers must endure. This shame and embarrassment stem not only from the fact that the sons steal from them but also from close friends and relatives. The participants described how they sometimes isolate themselves from the social engagement and community events because of their children’s persistent stealing. Some caregivers also indicated that their personal and social relationships with friends have been estranged leading to the loss of close friends whom hitherto had helped them and their children with basic needs such as food and clothing. The severity of these strained relationships may have left some caregivers feeling frustrated and ashamed.

### Personal challenges

The interviews revealed that feelings of panic and nervousness experienced by the caregivers arose primarily because of the enormous problems associated with their children’s behaviour. Panic attacks and nervousness are heightened due to the caregivers’ inability to seek help or talk to other people about their problems. It was however revealed that the thought of the existence of other children of theirs gave such caregivers hope and support to live. Fear of being on the receiving end of any bad news related to the sons’ addiction as well as the safety of other children were found in the narratives of the participants. The anticipation of adverse information due to their sons’ misbehaviour has been a traumatising experience for some caregivers.

Other participants blamed themselves for their sons’ reliance on methamphetamine. They attributed this to the fact that they might have given their children too much than they required. The use of words such as “spoiled him” and “I am to be blamed” reveals the extent to which self-blame for their sons’ behaviour was communicated. What was clear from the participants’ narratives was that, not only did they question their parenting skills but that they also wished they had done something different, and perhaps that they had set a good model for the younger children in the family.

### Family disruptions

Another effect that sons’ addiction had on caregivers was the disruption of family relationships. This disturbance in the family relations affects all primary members of the nuclear family (i.e. mother, father/and or step-father, and children). For some participants, these disrupted family relationships arose because of misunderstanding of the effect of methamphetamine use on the individual. For some participants, their husbands’ efforts in ensuring that cordial relationships existed in their family, might have been the genesis of troubled relationship in the family. It is plausible that the conflicting perspectives as to what constitutes an appropriate method to managing the sons’ addiction problems might have been a contributory factor. For some participants, their sons’ addiction to methamphetamine has exacerbated problems within the family, as the mothers found themselves constantly in conflict with their husbands, primarily arguing about their sons’ addiction. For some, such disruptions might have influenced various aspects of their lives as indicated by the use of the phrase “it has caused chaos”. For some participants, their sons’ aggressive behaviour was found to be unacceptable, leading to unpleasant altercations and strained relationships within the family, which have had some effect on other children in the family.

### Financial drain

Apparent in the experiences of the caregivers was financial burden associated with their sons’ behaviour. Some of the participants explained that their children’s stealing behaviour had not only eroded their economic resources but had also led to loss of the sons’ employment. Again, some participants also revealed that their sons used most of the income gained from a few hours of work solely for the purchase of methamphetamine, thereby reducing their contribution to the upkeep of their home.

## Discussion

Compared to previous studies on methamphetamine use within the Coloured communities in the Western Cape in South Africa, this study contributes towards building evidence on the experiences of caregivers whose sons are addicted to methamphetamine, a sample that has hitherto hardly been given much attention. By qualitatively examining these experiences, this study provides insight into the personal and interpersonal experiences associated with providing care for youth who are addicted to methamphetamine. Findings showed that methamphetamine-addicted youth misbehaviour provided a context that led to a troubled lifestyle for their caregivers. The consequences of caregivers’ sons’ misbehaviour is the feelings of shame and embarrassment. Participants also experienced personal challenges which included emotional problems, fear and self-blame. The findings also revealed that family disruptions and financial drain were other adverse experiences that are associated with their sons’ use of methamphetamine.

Several results from this study are consistent with other studies on mothers’ experiences of living with adolescents and/or youth substance abusers both in South Africa and beyond. For instance, the finding that some of the respondents experienced psychological distress because of their sons’ misbehaviour is consistent with other studies that have confirmed that repeated exposure to destructive behaviours on a daily basis affects the psychological well-being of mothers whose children have substance abuse problems [14, 21, 22]. A sense of worry could produce nervousness, and being nervous in a South African context has been found to be a risk factor for depression among caregivers [23]. Financial burden is known to be associated with substance addiction in young adults [9, 14, 24], as methamphetamine users most often sell everything in their possession including phones and clothes in order to pay for their drugs, thus leaving them with hardly any possessions [9]. Having disruptive family relations and financial burden may further exacerbate the psychological functioning of the caregivers, thus impacting adversely on the overall well-being of the family. This finding underscores the need for efforts to be directed at the development of formal support interventions for mothers of addiction-troubled youth.

The results also showed that the lifestyle of caregivers of youth addicted to methamphetamine is fraught with shame and embarrassment, which leads to a sense of isolation. This sense of isolation appears to facilitate perceived and expressed stigma towards substance users and their caregivers in South Africa [9, 15]. In the U.S. setting, studies have clearly shown that stigma and discrimination do not only discourage illicit drug users from getting health care due to fear of poor treatment by health care providers, but also affects the help-seeking behaviours of caregivers of adolescents with substance use problems [25, 26]. In this study, such behaviours might have prevented these mothers whose sons abuse methamphetamine from seeking out help and support from the broader social network, such as extended families and friends [9], often leaving them helpless and isolated. It is therefore important for interventions to put greater efforts into public education on the effects on stigma and the psychological distress associated with caregivers for young adults with substance addiction. Such efforts must also be directed at health care providers and formal treatment facilities to understand the challenges faced by parents and how best to provide support needed, as previous studies have suggested caregivers’ dissatisfaction with such services as they are most often misunderstood or blamed [14, 27].

The findings also showed that participants blamed themselves for their sons’ use of methamphetamine. The finding support the discourses which hold mothers accountable for their children’s behaviour [28, 29]. In this study, this accountability was illustrated in the ways the mothers blamed themselves, as caregivers, for their sons’ addiction to methamphetamine. This was implicit in the narratives where some of the caregivers questioned their own mothering approaches to understand why their sons are reliant on methamphetamine. In this way, the young adults’ addiction was inherently associated with the mothers’ happiness and sorrow. It has therefore been argued that mothers and/or caregivers of young adults with substance abuse problems often bear the burden of the child’s substance abuse and see the children as extensions of their own identity [29].

This study is not without some possible limitations. The researchers interviewed a purposive sample of women whose sons use crystal methamphetamine, so these findings may not reflect the experiences of caregivers whose children are addicted to other substances. As with all research of subjective experiences, there is also a possibility of recall bias. Additionally, this is a cross-sectional qualitative study, so the researchers are also unable to say if the experiences of the caregivers in the study resemble those of people with similar problems, all Coloured South Africans residing in Cape Town, or other social and economic groups in South Africa. However, the study provides useful insight into the lived experience of mothers whose children are addicted to crystal methamphetamine within a working class in South Africa.

## Conclusion

The findings from this study make a significant contribution to the understanding of the stressful and difficult experiences of mothers whose children are addicted to crystal methamphetamine within an environment characterised by crime, violence and high levels of unemployment. The study indicates that youth misbehaviour, caregivers’ personal challenges including emotional problems, self-blame and fear, were identified as critical themes in the lived experiences of caregivers. Several participants also indicated that financial drain and family disruptions were some of the keys issues that caregivers of youth addicted to methamphetamine experience. The importance of identifying and responding to the mental health needs of these families affected by methamphetamine cannot be overemphasised. In this regard, efforts should be directed at the development of more formal methods to provide the self-care skills for mothers of youth who are troubled by drug addiction. This could be achieved by equipping caregivers and parents with the appropriate skills to identify drug use in their children and how to respond to their children’s needs. This could perhaps be the first step towards curbing and gaining control over the negative impact of methamphetamine use in such communities.

## References

[1] United Nations Office on Drugs and Crime. World drug report 2016. Retrieved from https://www.unodc.org/wdr2016/.

[2] Pluddemann A, Flisher AJ, McKetin R, Parry C, Lombard C (2010). A prospective study of methamphetamine use as a predictor of high school non-attendance in Cape Town, South Africa. Subst Abuse Treat Prev Policy.

[3] Panenka WJ, Procyshyn RM, Lecomte T, MacEwan GW, Flynn SW, Honer WG (2013). Methamphetamine use: A comprehensive review of molecular, preclinical and clinical findings. Drug Alcohol Depend.

[4] Peltzer K, Ramlagan S, Johnson BD, Phaswana-Mafuya N (2010). Illicit drug use and treatment in South Africa: A review. Subst Use Misuse.

[5] Lane T, Raymond HF, Dladla S, Rasethe J, Struthers H, McFarland W, McIntyre J (2011). High HIV prevalence among men who have sex with men in Soweto, South Africa: Results from the Soweto Men’s Study. AIDS Behav.

[6] van Heerden MS, Grimsrud AT, Seedat S, Myer L, Williams DR, Stein DJ (2009). Patterns of substance use in South Africa: results from the South African stress and health study. S Afr Med J.

[7] Pluddemann A, Myers BJ, Parry CD (2008). Surge in treatment admissions related to methamphetamine use in Cape Town, South Africa: Implications for public health. Drug Alcohol Rev.

[8] Kapp C (2008). Crystal meth boom adds to South Africa's health challenges. Lancet.

[9] Watt MH, Meade CS, Kimani S, MacFarlane JC, Choi KW, Skinner D (2014). The impact of methamphetamine (“tik”) on a peri-urban community in Cape Town. South Africa Int J Drug Policy.

[10] Pluddemann A, Flisher AJ, McKetin R, Parry C, Lombard C (2010). Methamphetamine use, aggressive behaviour and other mental health issues among high-school students in Cape Town. South Africa Drug Alcohol Depend.

[11] Dada S, Pluddemann A, Parry C, Bhana A, Vawda M, Perreira T. Monitoring alcohol and drug abuse trends in South Africa, July 1996–December2011. In South African Community Epidemiology Network on Drug Use (SACENDU) Research Brief, 15(1):2012.

[12] Serra G, Warda S (2013). 1 in 5 Cape children on tik’.

[13] Parry CD, Myers B, Plüddemann A (2004). Drug policy for methamphetamine use urgently needed. S Afr Med J.

[14] Groenewald C, Bhana A (2016). “It was bad to see my [Child] doing this”: Mothers’ experiences of living with adolescents with substance abuse problems. Int J Ment Health Addict.

[15] Sorsdahl K, Stein DJ, Myers B (2012). Negative attributions towards people with substance use disorders in South Africa: Variation across substances and by gender. BMC Psychiatry.

[16] Meade CS, Watt MH, Sikkema KJ, Deng LX, Ranby KW, Skinner D, Kalichmann SC (2012). Methamphetamine use is associated with childhood sexual abuse and HIV sexual risk behaviours among patrons of alcohol-serving venues in Cape Town. South Africa Drug Alcohol Depend.

[17] Watt MH, Kimani SM, Skinner D, Meade CS (2016). “Nothing is free”: A qualitative study of sex trading among methamphetamine users in Cape Town, South Africa. Arch Sex Behav.

[18] Hobkirk AL, Watt MH, Myers B, Skinner D, Meade CS (2016). A qualitative study of methamphetamine initiation in Cape Town, South Africa. Int J Drug Policy.

[19] City of Cape Town Census (2012). The 2011 city statistics and population census.

[20] Braun V, Clarke V (2006). Using thematic analysis in psychology. Qual Res Psychol.

[21] Baskin-Sommers A, Sommers I (2006). The co-occurrence of substance use and high-risk behaviours. J Adolesc Health.

[22] Orford J, Velleman R, Natera G, Templeton L, Copello A (2013). Addiction in the family is a major but neglected contributor to the global burden of adult ill-health. Soc Sci Med.

[23] Casale M (2013). The protective role of social support for the health of caregivers of children in HIV-endemic South Africa.

[24] Swanson SM, Sise CB, Sise MJ, Sack DI, Holbrook TL, Paci GM (2007). The scourge of methamphetamine: impact on a level I trauma centre. J Trauma Acute Care.

[25] Ahern J, Stuber J, Galea S (2007). Stigma, discrimination and the health of illicit drug users. Drug Alcohol Depend.

[26] van Boekel LC, Brouwers EP, van Weeghel J, Garretsen HF (2013). Stigma among health professionals towards patients with substance use disorders and its consequences for healthcare delivery: Systematic review. Drug Alcohol Depend.

[27] Choate P (2011). Adolescent addiction: What parents need?. Procedia Soc Behav Sci.

[28] Butler R, Bauld L (2005). The Parents’ experience: Coping with drug use in the family. Drugs.

[29] Smith J, Estafan A (2014). Families parenting adolescents with substance abuse-recovering the mother’s voice: A narrative literature review. J Fam Nurs.

